# Simultaneous or sequential treatment of IgA nephropathy - specific disease drivers and non-specific consequences of nephron loss?

**DOI:** 10.1093/ckj/sfag035

**Published:** 2026-03-03

**Authors:** Jonathan Barratt, Jürgen Floege, Carmine Zoccali, Richard Lafayette

**Affiliations:** University of Leicester, UK; Department of Nephrology and Department of Cardiology, RWTH Aachen University Hospital, Aachen, Germany; Institute of Biology and Molecular Genetics (BIOGEM), Ariano Irpino, Italy; Renal Research Institute, New York, USA; IPNET, Via Torrione 42, 89125, Reggio Calabria, Italy; Division of Nephrology, Department of Medicine, Stanford University, Stanford, CA, USA

**Keywords:** IgA nephropathy, immunosuppression, nephron loss, supportive care, treatment strategy

## Abstract

The expanding therapeutic armamentarium for IgA nephropathy (IgAN) has brought to the forefront a key strategic dilemma: whether to intervene simultaneously on IgAN-specific pathogenic mechanisms and the downstream, non-specific consequences of nephron loss, or to follow a sequential, stepwise approach in which therapies are added only as residual risk becomes evident. This debate article contrasts these philosophies. Proponents of early, combined treatment, in line with KDIGO 2025 guidance, argue that the traditional paradigm of first maximizing ‘supportive’ care—renin–angiotensin system blockade, strict blood pressure and sodium control, and more recently SGLT2 inhibitors and mineralocorticoid receptor antagonists—before initiating immunosuppressive or other disease-directed agents embeds harmful delay. They emphasize that IgAN is driven by continuous immune activity, with ongoing production of pathogenic IgA, immune complex formation, complement activation, and glomerular injury that proceeds even when proteinuria is hemodynamically reduced. Once a critical threshold of nephron loss is crossed, self-perpetuating hyperfiltration, fibrosis, and microvascular rarefaction limit reversibility. Advocates of a more sequential strategy highlight heterogeneity in disease course, the risks and costs of polypharmacy, and the need for individualized escalation based on dynamic risk assessment. Together, the opposing yet complementary viewpoints underscore that future algorithms will likely integrate baseline risk stratification, evolving biomarkers of immune activity and chronic damage, and patient preferences to balance early, comprehensive disease control against overtreatment and toxicity in IgAN.

The emergence of multiple effective interventions for IgA nephropathy (IgAN) has sharpened, rather than resolved, a central strategic question: should we treat the disease by targeting IgAN‐specific pathogenic mechanisms and the consequences of nephron loss simultaneously from the outset, or should we proceed in a structured, sequential manner, adding therapies stepwise as residual risk becomes apparent? Herein, Jonathan Barratt and Juergen Floege, on one side, and Richard Lafayette, on the other, articulate these positions in a complementary and, in many respects, constructively opposing fashion. Together, they invite a synthesis that can inform contemporary clinical decision-making.

## SIMULTANEOUS TREATMENT: THE PRO POSITION

Advocates of simultaneous treatment, in keeping with the direction anticipated in the KDIGO 2025 guidance [1], argue that the traditional stepwise paradigm embeds harmful delay into the care of many patients. Historically, clinicians were taught to exhaust so-called ‘supportive’ measures first—renin–angiotensin system blockade, strict blood pressure control, sodium restriction, and other lifestyle measures, and more recently SGLT2 inhibition and mineralocorticoid receptor antagonism—before contemplating immunosuppressive or other IgAN-specific agents [2]. This was rational in an era in which disease—directed therapies were non-specific, prone to adverse events in particular infections, and often poorly tolerated. However, this approach tacitly assumed that IgAN would remain quiescent or at least indolent while supportive measures were being optimized. Mechanistic and clinical data suggest that this assumption is often false.

IgAN is now assumed to be a disease driven by continuous immunologic activity: production of galactose-deficient IgA1 and other pathogenic IgA forms, formation of circulating immune complexes, complement activation, and mesangial injury [3]. This pathogenic cascade persists even when proteinuria is attenuated by haemodynamic interventions [4]. Supportive therapies mitigate glomerular hyperfiltration and lower intraglomerular pressure, thereby reducing proteinuria; they do not, however, prevent immune complex formation and deposition. In this context, a strategy that waits for supportive care to ‘fail’ before introducing IgAN-specific therapy necessarily allows ongoing, largely invisible nephron injury during the waiting period. Once nephron loss crosses a critical threshold, the resulting hyperfiltration, tubular–interstitial fibrosis and microvascular rarefaction become self-perpetuating and only partially reversible, even if immunologic activity is later controlled. Observational cohorts and contemporary randomized trials consistently show that patients meeting classical trial inclusion criteria—proteinuria persisting above roughly 1 g/day despite what is deemed optimized supportive care—often experience substantial annual declines in eGFR way in excess of physiological aging [4]. In control arms of recent registration studies, annual eGFR ranged from -1.5 to -15 mL/min/1.73 m² despite best available supportive therapy, underscoring that haemodynamic control alone is frequently inadequate to prevent progressive nephron loss [4].

A further pillar of the simultaneous-treatment argument is the evolution of therapeutic targets. KDIGO 2025 [1] is set to articulate a more ambitious goal for IgAN: not merely slowing decline or partially reducing proteinuria, but approximating physiological eGFR loss rates in concert with very low, near-normal, proteinuria (i.e. below 0.3–0.5 g/day) [5]. Supportive modalities, even in combination, can rarely achieve these stringent targets in a majority of high-risk patients. RAS blockade typically yields a 30–40% proteinuria reduction, SGLT2 inhibition may add a further 20–30%, and MRAs can provide incremental benefit; yet significant residual proteinuria and hematuria often persist [6]. By contrast, IgAN-specific therapies—targeted-release budesonide acting at the mucosal immune interface, complement inhibitors targeting distinct steps in alternative or lectin pathways, and emerging B-cell directed strategies—address upstream drivers of disease biology. Early data suggest that these agents can, in a substantial subset of patients, reduce proteinuria to low-risk levels, normalise or nearly normalise the eGFR slope and in many cases abolish microhematuria [4]. Importantly, these newer agents tend to be more targeted and better tolerated than traditional systemic corticosteroids or cytotoxic drugs, shifting the risk–benefit balance in favour of earlier introduction.

Simultaneous treatment is also defended on pragmatic grounds. In many health systems, regulatory and reimbursement frameworks restrict access to IgAN-specific therapies to patients who exceed defined proteinuria thresholds, commonly ≥1 g/day or similar [6]. Effective supportive therapy can reduce proteinuria to just below those cut-offs without extinguishing immunologic activity. When this happens, a paradox arises: patients who may still have a reversible, pathogenetically active phase of disease become ineligible for drugs that target precisely that biology. The simultaneous-treatment paradigm seeks to pre-empt this problem by initiating IgAN-specific therapy in high-risk patients while proteinuria is still above regulatory thresholds, in parallel with intensive supportive care, thereby closing the window during which active disease would otherwise continue unchecked behind a façade of improved proteinuria [7].

Underlying these arguments is a coherent pathophysiological model. IgAN progression is conceptualised as a vicious cycle in which immune complex–mediated glomerular injury triggers compensatory and maladaptive CKD mechanisms—hyperfiltration, RAAS activation, fibrosis—which in turn exacerbate glomerular damage and nephron dropout. Simultaneously treating both the immunologic ‘ignition’ and the CKD ‘propagation’ mechanisms is an attempt to break this cycle early, before irreversible architectural damage dominates the clinical course. From this perspective, limiting treatment to supportive care for a prolonged initial period appears mechanistically and clinically misaligned with our current understanding of the disease.

## SIMULATENOUS TREATMENT: THE CON POSITION

In contrast, the case for sequential therapy, as articulated in Dr Lafayette’contribution, starts from a different set of clinical premises. IgAN is unquestionably a serious disorder, associated with a heightened risk of cardiovascular disease, reduced quality of life, kidney failure and premature mortality [8]. Yet the disease is heterogeneous. A non-trivial proportion of patients will not experience rapid progression, and some will maintain stable kidney function for decades. At the same time, the therapeutic armamentarium has become crowded and expensive, and long-term safety data for many novel agents remains incomplete. In this landscape, a default strategy of combining multiple interventions from the outset, particularly in unselected patients, may expose many individuals to unnecessary risks, costs and complexity.

Sequentialists emphasise the gains already achievable with a structured, stepwise approach anchored in rigorous risk stratification. When a recent biopsy is available, the International IgAN Prediction Tool [9] provides a means of integrating histologic and clinical features into an estimate of individual risk; where histology is unavailable or outdated, proteinuria, blood pressure and eGFR trends remain robust, pragmatic markers. Therapeutic goals—tight blood pressure control, substantial reduction of proteinuria to low-risk or ideally normal levels and stabilisation of kidney function—are viewed as achievable for many patients without immediate resort to multi-drug combination strategies. Recent and historical data demonstrate that monotherapy or dual therapy with agents that primarily target generic mechanisms of kidney injury can substantially alter the disease course. Classic enalapril studies in young patients with preserved GFR and proteinuria around 2 g/day showed that RAAS blockade alone could reduce proteinuria by more than half on average and improve long-term kidney survival dramatically, with more than 90% of treated patients free from a 50% increase in serum creatinine at seven years compared with roughly half of controls. More contemporary trials have shown that selective antagonists of the endothelin receptor [10] and dual endothelin–angiotensin receptor antagonists [11] and SGLT2 inhibitors [12] can independently slow eGFR decline and increase rates of partial or complete proteinuria remission across broad IgAN populations, independent of specific biopsy features or baseline eGFR strata.

Similarly, agents considered ‘anti-inflammatory’ or disease-specific—systemic steroids [13], targeted-release budesonide [14], complement inhibitors such as iptacopan [15]—have not, to date, been shown to benefit only patients with overtly inflammatory or early-stage disease. On the contrary, trial populations have often included individuals with predominantly sclerotic lesions and reduced GFR, and yet these agents have still conferred reductions in proteinuria and improvement in risk trajectories. This argues against our current ability to precisely match particular drug classes to narrow histologic or clinical phenotypes and undercuts simplistic algorithms that would reserve one class exclusively for ‘inflammatory’ disease and another for ‘sclerotic’ disease.

Within this evidentiary context, the sequential approach proposes a pragmatic, patient-centred pathway. Following diagnosis, early and comprehensive risk assessment should be performed. Unless the patient presents with very aggressive disease—approaching rapidly progressive glomerulonephritis—it is reasonable to initiate a first-line intervention and then allow sufficient time, typically on the order of three to four months, to assess its effect on proteinuria, blood pressure, eGFR trajectory and overall risk. If the risk profile remains unacceptably high, the clinician can either switch to an alternative strategy (if the first agent appears ineffective or poorly tolerated) or add a second agent (if the initial therapy is working but insufficiently). Another cycle of treatment and re-assessment follows, again over a few months. In this way, individual drugs are evaluated for both efficacy and tolerability in the specific patient, and escalation is guided by demonstrable residual risk rather than by theoretical concern alone.

Sequentialists acknowledge the concern that delaying the initiation of all potentially relevant therapies might expose some patients to avoidable nephron loss. However, they note that IgAN, even when progressive, usually takes months to years to meaningfully impact GFR. In their view, carefully monitored therapeutic trials over a few months, in which response and risk are re-evaluated iteratively, are not only ethically defensible but, in many cases, optimal. Importantly, there is compelling evidence that high-risk patients can move into a low-risk category with a single additional intervention on top of baseline RAAS blockade—for example, switching to a dual endothelin–angiotensin receptor antagonist or adding an IgAN-targeted agent such as Nefecon or iptacopan, which have been associated with marked increases in the odds of achieving low-risk proteinuria thresholds. If many patients can be adequately protected by one or two sequentially chosen therapies, the universal adoption of simultaneous multi-agent regimens risks overtreatment.

From a safety and economic perspective, the sequential strategy also has appeal. Introducing one new therapy at a time allows clearer attribution of adverse events, simplifies pharmacovigilance and minimises the risk of unrecognised synergistic toxicity. It allows clinicians to identify ‘super-responders’ to individual interventions and to avoid layering on additional therapies whose marginal benefit in that specific patient might be small. In an era of high drug costs and constrained healthcare resources, such a tailored, parsimonious approach carries weight.

## THE MODERATOR’S VIEW

**Figure 1:**
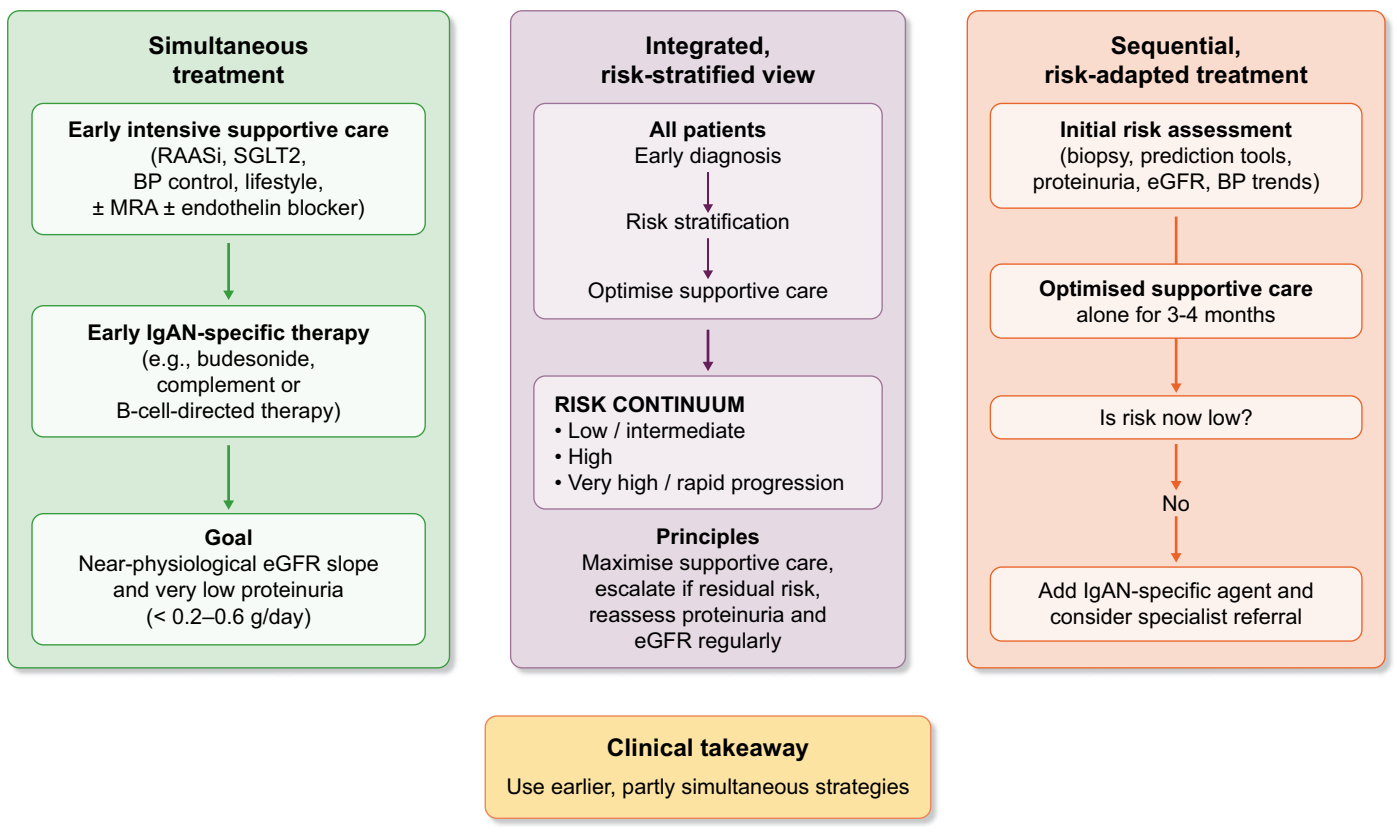
The figure compares three therapeutic strategies for IgA nephropathy. The left column illustrates simultaneous treatment, in which intensive supportive care with RAAS inhibition, SGLT2 inhibition, blood pressure control, lifestyle measures and, where appropriate, mineralocorticoid receptor antagonists or endothelin blockers is started early together with IgAN–specific therapy such as budesonide, complement inhibitors or B–cell–directed agents; the goal is to achieve a near–physiological eGFR slope and very low proteinuria, thereby interrupting the vicious cycle of kidney damage as early as possible. The right column represents a sequential, risk–adapted strategy: an initial comprehensive risk assessment based on biopsy findings, prediction tools, proteinuria, eGFR and blood pressure trends is followed by a period of optimised supportive care alone; after 3–4 months, risk is re–evaluated, and if it remains high, IgAN–specific therapy is added and specialist referral is considered, whereas if risk is low, the current supportive regimen is continued. The middle column shows an integrated, risk–stratified approach in which all patients undergo early diagnosis and formal risk stratification, followed by optimisation of supportive care; patients are positioned along a risk continuum from low or intermediate risk to high and very high or rapidly progressive disease, and treatment is escalated if residual risk remains, with regular reassessment of proteinuria and eGFR slope to avoid both undertreatment and overtreatment. The clinical takeaway is that high–risk or rapidly progressive IgA nephropathy generally calls for earlier and at least partly simultaneous use of supportive and disease–specific therapies, while lower–risk disease may be managed with a more sequential, stepwise approach guided by repeated risk reassessment.

Viewed through a moderator’s lens, these two positions are less mutually exclusive than they initially appear. Both recognise that IgAN is a chronic, immunologically mediated kidney disease with superimposed generic CKD processes; both accept that supportive care is necessary but often insufficient, and both acknowledge the promise and limitations of new IgAN-specific agents. The disagreement is primarily about timing, intensity and generalisability of combination therapy (Fig. 1).

An integrated, practice-oriented view might proceed along the following lines (Fig. 1). First, early and accurate risk stratification should be a non-negotiable cornerstone of care. Whenever possible, this includes a contemporary biopsy and use of validated prediction tools, supplemented by close attention to proteinuria magnitude and trajectory, blood pressure control and eGFR trends. Second, optimal supportive therapy must be initiated promptly in all patients: maximally tolerated RAAS blockade, strict blood pressure targets, dietary and lifestyle counseling and, where appropriate, SGLT2 inhibition and other CKD-protective agents. This is one area of solid consensus.

Third, the decision between simultaneous and sequential addition of IgAN-specific therapies should be individualised based on baseline risk, speed of progression and regulatory context. In patients with clear high-risk features at presentation—for example, sustained heavy proteinuria, rapid eGFR decline, adverse histologic markers or a high-risk prediction score—and in jurisdictions where IgAN-specific therapies are accessible once predefined proteinuria thresholds are met, there is a strong mechanistic and clinical rationale for relatively early combination of supportive care and at least one disease-specific agent, rather than deferring the latter for prolonged periods. In these patients, the potential for silent, irreversible nephron loss during months of ‘waiting to see’ is high, and the safety profile of modern targeted therapies makes early intervention acceptable.

Conversely, for patients whose risk appears intermediate or modest, and for those in whom supportive therapy leads to substantial, rapid and stable reductions in proteinuria and normalization or near-normalization of risk estimates, a more sequential approach is justified. In such individuals, the introduction of additional, expensive or potentially risky therapies should be contingent on demonstration of residual risk or inadequate response. Systematic re-assessment at predefined intervals, perhaps every three to four months initially, allows clinicians to detect those who are not achieving low-risk profiles and to escalate treatment in a timely yet measured fashion. For many, a small number of well-chosen interventions, introduced and evaluated in sequence, may suffice to achieve durable risk reduction.

Across the spectrum of risk, it is crucial to avoid two pitfalls. The first is rigid adherence to a ‘supportive-first, disease-specific later’ algorithm that ignores accumulating evidence of early eGFR loss under supportive monotherapy in a subset of patients. The second is an uncritical embrace of ‘more is better,’ in which multiple IgAN-specific and CKD-targeted agents are routinely combined from the outset without clear individualised justification, exposing patients to avoidable harm and health systems to unsustainable costs. The most defensible middle path is a dynamic one: start comprehensive supportive care without delay; assess risk carefully; in high-risk or rapidly progressive cases, move early to add at least one IgAN-specific therapy in parallel; in others, build therapy stepwise with close monitoring, ready to intensify without hesitation when residual risk remains.

In clinical practice, this translates into a commitment to personalised, data-driven care. Patients should be counselled that IgAN is neither uniformly benign nor uniformly catastrophic, that many new treatments are available but their long-term roles are still being defined, and that their care plan will likely involve cycles of intervention and reassessment. For the clinician, it demands familiarity with both the strengths of supportive therapies and the evolving evidence base for IgAN-specific agents, as well as an awareness of local regulatory thresholds that may inadvertently shape the timing of intervention.

Ultimately, the debate between simultaneous and sequential treatment in IgAN is best resolved not by dogma but by flexible application of both philosophies. Simultaneous treatment principles should guide the management of those at highest risk or with clear evidence of rapid progression, where time lost is kidney lost. Sequential treatment principles should temper the urge to overtreat lower-risk individuals, ensuring that each therapy is given a fair trial and that escalation is justified by persistent risk rather than theoretical possibility. As more long-term data accrue, the balance between these strategies will likely be refined. Until then, a nuanced, patient-centred synthesis of both approaches offers the most responsible course for contemporary clinical practice.
